# Methods for Patient-Centered Interface Design of Test Result Display in Online Portals

**DOI:** 10.5334/egems.255

**Published:** 2018-06-26

**Authors:** Daniel T. Nystrom, Hardeep Singh, Jessica Baldwin, Dean F. Sittig, Traber D. Giardina

**Affiliations:** 1 Department of Biomedical Informatics, University of Utah, US; 2 Center for Innovations in Quality, Effectiveness and Safety, Michael E. DeBakey Veterans Affairs Medical Center, and Department of Medicine, Baylor College of Medicine, US; 3 School of Biomedical Informatics, University of Texas Health Science Center, US

**Keywords:** health information technology, patient portal, electronic health record

## Abstract

**Objectives:**

Patients have unique information needs to help them interpret and make decisions about laboratory test results they receive on web-based portals. However, current portals are not designed in a patient-centered way and little is known on how best to harness patients’ information needs to inform user-centered interface design of portals. We designed a patient-facing laboratory test result interface prototype based on requirement elicitation research and used a mixed-methods approach to evaluate this interface.

**Methods:**

After designing an initial test result display prototype, we used multiple evaluation methods, including focus group review sessions, expert consultation, and user testing, to make iterative design changes. For the user testing component, we recruited 14 patient-users to collect and analyze three types of data: comments made during testing sessions, responses to post-session questionnaires, and system usability scores.

**Results:**

Our initial patient-centered interface design included visual ranges of laboratory values, nontechnical descriptions of the test and result, and access to features to help patients interpret and make decisions about their results. Findings from our evaluation resulted in 6 design iterations of the interface. Results from user testing indicate that the later versions of the interface fulfilled patient’s information needs, were perceived as usable, and provided access to information and techniques that facilitated patient’s ability to derive meaning from each test result.

**Conclusions:**

Requirement elicitation studies can inform the design of a patient-facing test result interface, but considerable user-centered design efforts are necessary to create an interface that patients find useful. To promote patient engagement, health information technology designers and developers can use similar approaches to enhance user-centered software design in patient portals.

## Background and Significance

Timely access to high-quality, accurate, easy to interpret, patient-specific clinical data and reference information is a fundamental component of any clinical decision support system (CDSS) [1]. Patient access to their health information via portals proposes to increase patient engagement in the clinical decision-making process and thus improve quality and safety of care [2]. By providing direct access to health information, patient portals have improved patient engagement [3] and empowered patients to take control of their care by giving them a more informed perspective of their health [4].

Despite the benefits provided by patient portals, few patients use them [5]. In particular, research has suggested that patients have difficulty interpreting laboratory test results that are communicated via online channels (patient portals, emails, etc.) [6]. Our recent study suggested that current portals are not designed to present test results to patients in a patient-centered way and patients often experienced negative emotions with abnormal results and sometimes even with normal results [7]. We concluded that simply providing access is insufficient; this must be accompanied by design strategies to help patients interpret and manage their test results. Another study identified the source of this problem as patients’ lack of medical knowledge, which constrains their ability to comprehend concepts required to form good decisions [8]. However, the issue of misunderstanding or misinterpreting test results can also be framed as a lack of appropriate interface design that fails to provide patients with the necessary visual cues and reference information they need to comprehend each test result to make informed decisions.

The effective use of user-centered design principles and methodologies has started to gain momentum for clinician-facing applications of electronic health record (EHR) systems [9,10]. Though there has been mention of the importance for user-centered design when developing patient-facing applications within EHRs [11] (such as patient portals), few studies have managed to carry-out the user-centered design process [12] in its entirety. The majority of studies on patient-centered design have explored patients’ information needs when interpreting their test results and how appropriate informatics tools can improve patient comprehension of health concepts [13,14,15,16,17]. However, few published studies have attempted to take the next steps to use these information requirements to propose new designs for patient-facing interfaces or tools that promote patients’ comprehension of test results and to test these interfaces with patients [16].

## Objectives

In this study, we used findings from previous research eliciting patient information needs for understanding their laboratory test results to inform the design of a prototype laboratory test result review interface. After creating the initial prototype, we describe the procedure used to iteratively evaluate and test the new user interface. We describe the methods in detail so that it can be used and/or adapted by others to inform better user-centered design for patient portals. While one of the primary purposes of this work is to inform the design of an interface to display test results to patients in ways they can comprehend, we did not determine actual effects of the interface on patient comprehension of test results. Rather than emphasizing comprehension – implying a measure of “correctness” of each patient’s interpretation of each test result – we focused on patient’s ability to generate meaning from each test result and how the meaning they generated would inform their decision making and subsequent actions.

## Design Considerations

One of our main considerations to drive the design of the interface was the desire to make the system “usable” by patients. We used available literature [4,13,18,19,20,21,22,23,24] to influence the usability of the prototype system during initial development, and then addressed additional usability concerns discovered through our evaluation strategy which included expert consultations, focus-group review sessions, and user testing sessions. User testing sessions collected three measures from patient participants to directly assess the usability of the prototype test results interface. First, comments and suggestions made by patients during user testing sessions helped identify ways to improve future versions of the interface. Second, the System Usability Scale (SUS) [25] – a questionnaire used to estimate a product’s usability – was used to quantify usability and allow for a more objective comparison of usability across different versions of the prototype. Third, a post-session questionnaire was developed to collect patient’s overall perceptions of the interface.

The second consideration that drove the design of the prototype test result interface was to encourage patient’s ability to “make sense” [26,27] of each test result. Klein, Moon, and Hoffman define sensemaking as “a motivated, continuous effort to understand connections in order to anticipate their trajectories and act effectively” [26,27]. In the current work, we attempt to design an interface that facilitates connections between patient’s current knowledge of test results, the physiology that is captured by each test result, and what patient’s would anticipate doing after receiving these test results. Although sensemaking is not explicitly measured in our evaluation, comments made by patients during sessions were used to draw insight into their understanding of the test results displayed in the prototype given the context of a case vignette. An additional proxy measure of patient sensemaking was patient responses to questions on the post-session questionnaire that was administered after each testing session. Specific questions on the post-session questionnaire were tailored to determine if the information identified by requirement elicitation studies were sufficient to address patient information needs to derive meaning for each test result.

## System Design

To assist with designing the test result interface, we referenced a process map that depicts the patient perspective of the test result process [23] and additional literature that elicited patient information needs and requirements to comprehend test results [4,13,18,19,20,21,22,23,24]. Since most adult patients are likely to have a lipid profile (i.e., a group of laboratory tests of a patient’s blood to identify various levels of fat and fat-like substances such as cholesterol) drawn at some point, we chose to base our initial interface design on lipid profile test results. In addition to lipid profile test results, we also created displays for laboratory tests that are not based on a range of values (hepatitis B surface antigen test results) and created a display for a test result that participants are likely unfamiliar with (liver function test results) that were included in later versions of the prototype.

**Figure 1:**
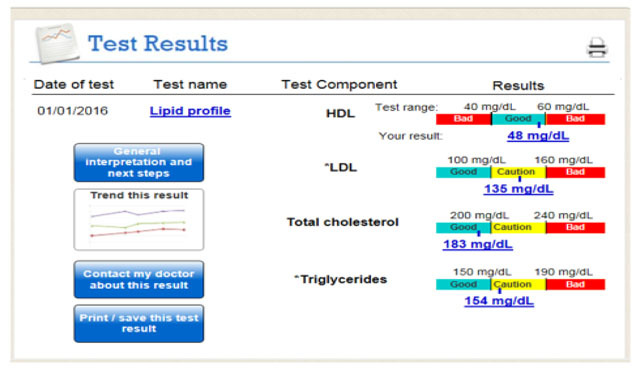
Initial patient-centered lipid profile test result interface accounting for patient’s needs and preferences for understanding laboratory test results.

After collecting patient information needs and requirements from previous literature, we used Axure® [28] prototyping software to generate an html version of the prototype test result interface. Our goal was to make the needed information easy to detect and interpret by using graphical representations [20,24] and short, non-technical verbiage to describe medical concepts. The interface was designed to facilitate the use of features that might help patients interpret their test results, such as providing links to reputable internet resources where they could learn more about their test result [8,19]. Figure 1 shows a screenshot of the initial lipid profile test results review interface accounting for patient’s preferences and needs to understand laboratory test results.

Clicking a button or hyperlinked text in the display will generate a pop-up window that contains information pertaining to the selected button/hyperlinked text. Although the use of “pop-ups” as a method to present information was not specifically identified in the literature, we anticipated that pop-ups would ease navigation of the interface by allowing patient-users to always be able to identify the test result “home screen” (i.e., Figure 1). For more description of each button and hyperlinks’ functionality and a list of the requirement elicitation studies that informed the information presented by each button/hyperlink in the prototype test result interface, see Appendix 1.

## Iterative Prototype Refinement and Evaluation

We used a multimethod formative evaluation approach involving multidisciplinary review sessions, expert consultations, and usability testing to uncover additional patient information needs and determine patient satisfaction with the test result interface. During the iterative evaluation and re-design phase we incorporated principles and suggestions from relevant interface design literature to improve the usability of the test result interface [29,30].

The primary goal of each usability evaluation phase was to enhance the interface functionality by iteratively incorporating findings from the evaluation and usability testing sessions. To achieve the goal of iterative interface design, the methods used to evaluate the prototype test result interface were not conducted sequentially, rather these methods were used synergistically. The multidisciplinary review sessions occurred during early versions of the interface design, but the expert consultations and user testing sessions shared significant overlap on the design timeline. All portions of the evaluation phase of this research were approved by our local Institutional Review Board.

### User testing

The usability test required patients to use the prototype test result interface to navigate to and interpret three different test results: an abnormal lipid profile test result, a hepatitis B surface antigen test result that suggested getting an immunization, and a liver function test that was within normal limits.

### Participants

To recruit participants for the usability portion of the design evaluation, we contacted patients who identified themselves as open to participating in a related research project [31] via email. Patients who consented to participate in this research were offered a $25 dollar gift card upon completion of their testing session. To avoid skewing the perceptions and results of the later versions of the test result interface, participants were only invited to use one version of the interface and were not allowed to participate again after their initial testing session.

### Equipment and Software

All testing sessions took place in either a private room in the researcher’s office or the patient’s preferred location. Testing was conducted using a laptop computer and computer mouse to ease navigation of the computer interface.

For each testing session, Axure was used to generate an html-based interface for participants to navigate during the testing session. Camtasia software [32] – a screen capture and audio recording software that runs in the background of the device that is being used – was used to capture participants’ cursor behavior while using the prototype and to obtain an audio record of verbal comments, questions, and concerns participants made about the prototype during the testing session [33,34].

### Procedure

Before beginning the testing session, participants were informed that the purpose of this study was an initial usability evaluation of a prototype patient portal laboratory test result interface and that this study involved audio and screen capture recordings. Participants were also informed that the laptop was connected to the internet and they were encouraged to use this resource to learn more about their test results if they felt the need to do so.

After consenting to participate, a vignette (Appendix 2) was read to patients to provide context for why they are using the prototype and to outline the task they needed to accomplish during the testing session. During the testing session, participants were asked to “think-aloud” [35] while navigating the display by periodically reporting what they were looking for, anything that surprised them about the interface, or anything that violated what they expected to find.

### Post-session interview and questionnaires

After finishing the testing session, participants were asked to fill out two questionnaires. First, a brief post-session questionnaire was created to query patients about what they would do after viewing the test results, their desire to learn more about each test result, and their overall experience using the prototype test result interface. Second, participants were asked to judge the usability of the interface using the System Usability Scale [25]. After completing the questionnaires, participants were asked if they had any final questions, comments, or concerns about the interface or the testing session.

## User Testing and Iterative Design

Fourteen patients volunteered to participate in the usability portion of this project. Participant ages ranged from 25 to 73 years with an average age of 43 years. Most patients had some experience working in the health care domain (71 percent). Occupations varied from less-clinically oriented (e.g., research project managers) to more clinically oriented professions (e.g., registered nurse). All participants indicated they had some familiarity with the tests presented in the test result interface.

**Figure 2:**
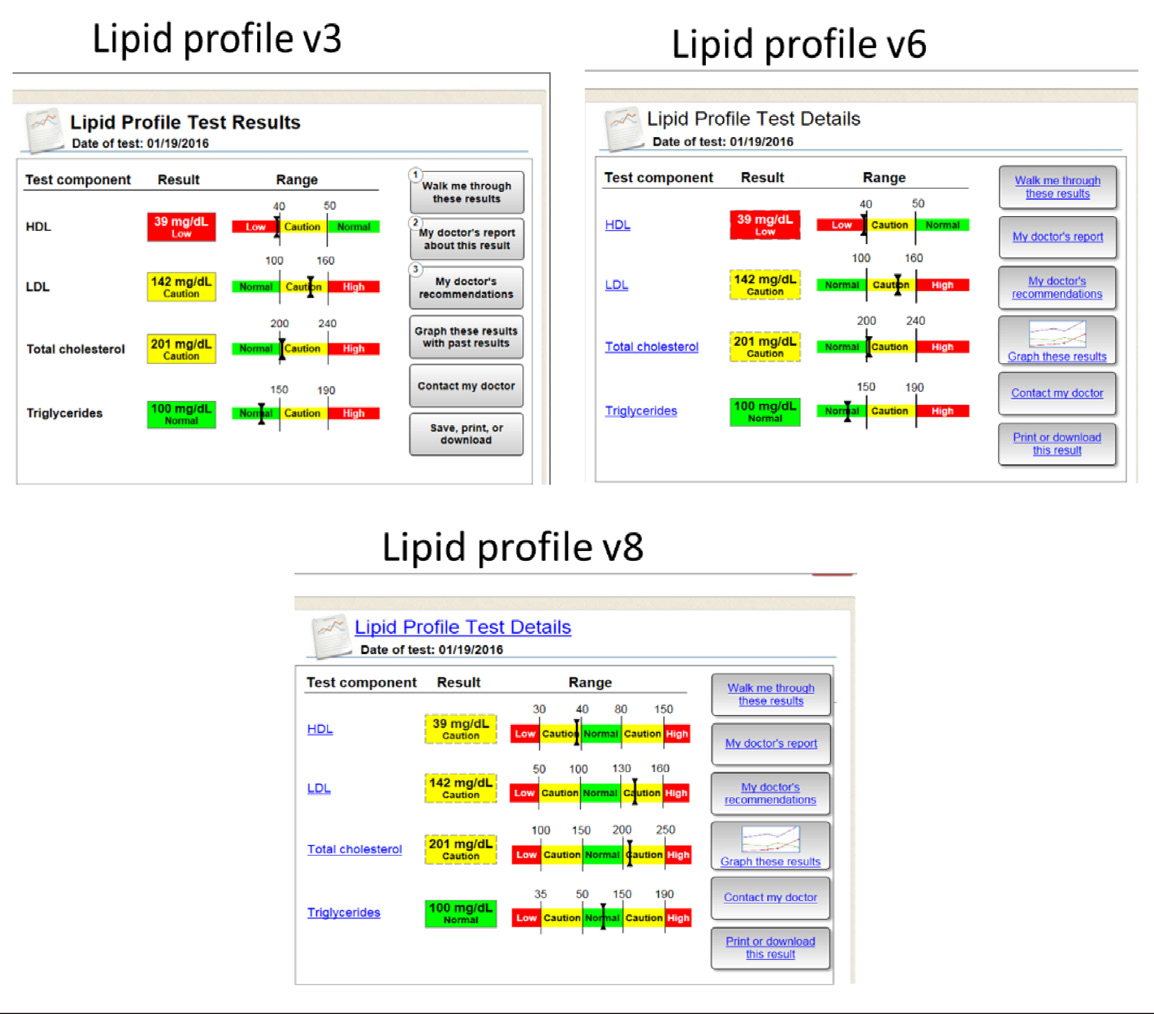
Screenshots of Version 3, 6, and 8 of the lipid profile test in the patient-centered test results interface.

To help interpret results of the usability evaluation and to illustrate the impact of feedback on the design of the patient-centered test result interface, three screen shots of different versions – Version 3, Version 6, and Version 8 – of the lipid profile test result interface are provided in Figure 2. Each subheading in the following results will discuss findings relevant to the three versions of the patient-centered test result interface.

### Design Process

Between each testing session, each version of the test result interface underwent considerable changes based on feedback provided by participants in previous usability testing sessions and prior results of the evaluation phase. Table 1 includes examples of iterative changes made to the prototype related to the lipid profile test result.

**Table 1:** Lipid Profile changes made based on user response examples.

Study ID	Prototype Version	User issues identified	Average SUS score for each version	Changes that were implemented

3002	3	– Didn’t realize test components were clickable – Didn’t understand ranges well	65	– Addressed clickability by making components more obviously clickable (i.e.; added highlighted circle around result to encourage clicking, and “hover over” cues, Dotted lines placed around Result box totals) – Caution range added for HDL result
3003	4	– Clicked on “walk me through these results” to learn more about triglycerides, but didn’t click on view detailed results at the end of test description – not apparent it will lead somewhere else other than main result page – Didn’t know to click on test components to learn more about the component	95	– Link walk through to detailed results, not test description – Make components more clickable
3004	5	– Didn’t see a way to “go back” from test explanation	92.5	– Created a “back arrow” button that returns to the main display page
3006	6	– Confused about HDL having low range in red – initially thought “oh HDL is low, that’s good” which is incorrect because it’s supposed to be on the higher range. Trend in other ranges is that high is bad except with HDL	85	– Add additional low and/or high to equalize all ranges on the display
3011	7	– Labels inside of ranges are on the lower end of readability	96.3	– Increase size of label font and range boxes – use heavier font

To determine if the information needs and requirements from relevant literature satisfied patients’ needs in the design of the patient-centered test result interface, results from the post-session questionnaire and SUS scores were analyzed. When asked if they thought any information was missing from the display, 21 percent of participants indicated that they wanted more information about factors related to their test results, such as facts about medication options and more details about each potential disease. Similarly, 36 percent indicated they would like to “Google” their results to retrieve relevant information about each result. When asked what they would search for on the internet, two participants who tested early versions of the prototype (Version 2 & 3) wanted to know more information about the clinical details of their test results: *“Yes. Triglycerides and albumin – trying to figure out what they are and how they affect the test.”* (P3003)

In later versions of the prototype (>Version 5), participants were less concerned with using the internet to understand the details of their test results. Instead, two participants wanted more information about cholesterol medications and the hepatitis B immunization mentioned within the test result interface. Another participant indicated that they would like to know what the average values of each test result are for “someone like me”: *“If they were my test results, I might want more information about what’s normal for someone like me – google “normal cholesterol or LFT” for someone like yourself.”* (P3009)

When asked what they would do after viewing the test results, 64 percent of participants mentioned they would make some form of lifestyle change, such as adjusting their diet, exercising more or exploring therapeutic options such as starting cholesterol medication or getting a hepatitis B vaccine. In addition, 35 percent of participants anticipated that they would contact their physician to hear their interpretation of the test results and/or discuss potential treatment options.

### SUS Scores

After completing the testing session, participants were asked to judge the usability of the prototype test result interface by filling out the SUS. Table 1 presents data on the average SUS score for each version of the interface. Every iteration following version 3, resulted in a SUS score of 82 or higher; suggesting the interface maintains an “acceptable” rating of usability according to Bangor, Kortum, and Miller’s evaluation of how to interpret the SUS [36].

## Discussion

In the current paper, we gathered patient information needs for understanding laboratory test results that were previously identified in the literature. We used these information needs to create a patient-centered interface that allowed patients to review a lipid profile, hepatitis B surface antigen, and liver function test result. The initial test result interface (Figure 1) primarily focused on providing the information needs identified in the literature with minimal consideration for the design of how this information would be presented to patients. After developing the initial interface, we used a variety of methods – multidisciplinary review sessions, expert consultations, and user testing – to evaluate and enhance the design of the interface. During early iterations of the design, expert opinions from clinicians ensured validity of the descriptions provided in the interface (e.g., when writing descriptions for the relationship between test result biomarkers and physiology) and to phrase descriptions in a way that eased readability but maintained accuracy. Focus group review sessions pooled expertise from a variety of fields including health care, computer science, and human factors engineering to generate an informed, highly-useable prototype to begin user testing.

Despite our best efforts, once user testing with patients began, numerous design modifications were discovered that were not considered or noticed by our team in earlier evaluations; a testament to the value of including users in the design process. For instance, one finding/design change that came exclusively from user testing was the design of the graphical range for the lipid profile test result. Initially, the range provided on the HDL portion of the lipid profile test result only showed the lower side of an HDL range (see Figure 2, Version 3 and Version 6), since low HDL levels have been associated with poor health outcomes. After testing this range with multiple patients, we discovered that patients were confused as to why the range for HDL was “the opposite” of the other ranges (LDL, total cholesterol, and triglycerides). Based on this feedback, we adjusted all of the ranges of the lipid profile display so they appear equalized across each biomarker (see Figure 2, Version 8). An unanticipated consequence of this design adjustment was the creation of non-linear scales, where the visual space for each biomarker are radically different than the other. We did not make any attempt to assess the impact of these non-linear scales on patient perceptions. Despite this lack of linearity, patients no longer identified the visual ranges as concerning in subsequent testing. This finding suggests patients are not concerned with the exactitude or linearity of each range, but are more concerned with where each result fits according to the range descriptors. Future research should explore patient perceptions of linearity and range descriptors for viewing laboratory test results.

Finally, the importance of user-centered design can be observed when comparing early versions of the patient-centered test interface to later versions of the interface (see Figures 1 and 2). The content being presented – that was derived from needs assessment literature – was relatively unchanged between these versions, but considerable effort was dedicated to making design features less confusing (e.g., Figure 2; HDL scale) and easier to navigate (e.g., Figure 2; improving the “clickability” of buttons by making them look like hyperlinks) [30]. In other words, simply providing information that has been identified from patient needs assessments and requirements studies (as seen in Figure 1), does not provide an adequate foundation for patients to make informed decisions. Rather, considerable effort is required to create an interface that frames this information in a way that allows patients to derive meaning from each result.

We anticipate the benefit offered by rigorous design to create an easy-to-use interface for patients to view laboratory test results allows patients to devote more of their attention to making sense of their test results, instead of being distracted by usability issues or having to visit other sources to collect more information about their test results. Patients who used later versions of the prototype were less concerned with collecting information about each test result and were more concerned with collecting information about what to do after receiving these test results. Future research should expand on this preliminary finding to more thoroughly explore the relationship between patient’s use of patient portals, the perceived usability of the portal interface, and patient’s understanding of the information presented in the portal.

## Limitations

Aside from a low sample size, the main limitation of the user testing portion of our evaluation is the lack of a homologous “stimuli” to test with participants. In the current study, we chose to implement a semi-structured design process that refrained from using a strict schedule to determine when to begin testing and making interface improvements during the design of the interface. This methodology increased the speed at which the interface could be produced at the cost of rigorous experimental design. Due to the limitations imposed by the design of the current study, the value added by this research is in the explicit description and illustration of methods that explain how patients’ information needs and requirements were incorporated into the design of an interface, and how the evaluation and testing of the interface enhanced its functionality.

An additional limitation that may affect the generalizability of the results of this study is the large percentage (71 percent) of patients who had history of working in health care prior to participating in this study. In addition, the use of red/green color schemes for the design of our interface could potentially limit the ease of using this interface for colorblind patients. While to our knowledge none of the test subjects were red/green color blind, we should note that “color” was not the only indicator that subjects had available to help them understand the meaning of the graphs or displays. As shown in Figure 2, dotted lines, bolding of text, and thick bars to denote boundaries within the range for each lipid result help to convey information similar to that indicated by color. Nevertheless, considering the purpose of this study – illustrating the use of information needs and user-centered design to create a patient-centered test result review interface – we do not recommend generalizing any of the preliminary results of this study that describe patient’s ability to interpret or comprehend test results. Rather, the value of this study is in its illustration of the methods and process we used to design and evaluate the prototype test result interface.

## Conclusions

Rigorous methods are needed to operationalize patient’s information needs and requirements to inform the design of patient-facing interfaces; a required first step in any clinical decision-making process. This study highlights how information from information needs and requirements elicitation studies could inform the design of a patient-facing, prototype test result interface and how user-centered design is necessary to make the findings of information needs studies useable by patients. Results of the current study suggest that patients perceive the interface as usable and fulfilling for most of their information needs when it comes to interpreting certain types of test results. This approach illustrates how patient-centered software could be better designed through findings from requirement elicitation and/or needs assessment studies. Health information technology designers and developers can use similar approaches to enhance user-centered software design in patient portals in order to promote patient engagement.

## Human Subjects

The local IRB committees at each site approved this study.

## Additional Files

The Additional files for this article can be found as follows:

10.5334/egems.255.s1 Appendix 1 General interface dynamics, button functionality, and hyperlink descriptions.

10.5334/egems.255.s1 Appendix 2 Case vignette provided to patient participants during usability testing.

10.5334/egems.255.s1 Appendix 3 Post-session questionnaire.
