# Supplementary material for: Methods for Patient-Centered Interface Design of Test Result Display in Online Portals
Source: EGEMS (Wash DC). 2018 Jun 26;6(1):15. doi: 10.5334/egems.255 (PMC6078112; doi:10.5334/egems.255)
Supplement: Appendix 1 — General interface dynamics, button functionality, and hyperlink descriptions. [file egems-6-1-255-s1.pdf]

Appendices

Appendix 1

Descriptions of button and hyperlink functionality and references to the requirement elicitation studies that informed the information presented in the initial patient-centered test result interface design (Figure 1). For each description, statements have been provided to help reference which part of the screen shown in Figure 1 is being discussed.

General interface dynamics, button, and hyperlink description

General interface dynamics

In general, clicking a button or hyperlinked text in the display will generate a pop-up window that contains information pertaining to the selected button/hyperlinked text.

"Lipid profile" hyperlinked text

Clicking this hyperlinked text opens a new tab in the internet browser that navigates to a reputable website[37] that contains more information about lipid profile tests.[4,19,22-24]

"General Interpretation and next steps" button

Clicking this button generates a pop-up window that contains a doctor-authored note that explains the test result's significant findings and provides recommendations on what the patient should do next (e.g., schedule a follow-up appointment, change health habits, etc.).[13,18,21,23]

"Trend this result" button

Clicking on this button generates a pop-up window that allows the user to compare test results within a selected date range.

"Contact my doctor about this result" button

Clicking this button opens the standard messaging page found within the EHR that allows a patient to type and send a message to their provider. However, since the messaging page was accessed via the button provided in the lipid profile test result page, the patient's message is auto-populated with details of the test result (including the date the result was drawn and significant findings) to assist the physician with identifying the test result the patient is referring to in their message.[18,19,21-24]

"Print / save this test result" button

Clicking this button generates a .pdf file of the test result that can be printed or saved for the patient's records. Clicking on the "printer" symbol located in the top right area of Figure 1 generates the same .pdf as the button on the lower-left side of the screen.[18,20,23]

Test components (HDL, LDL, Total cholesterol, Triglycerides)

Each of the lipid profile test components found on the right-side of Figure 1 carry the same three functions. First, the graphical range for each component helps identify where the patient's test result is located according to a reference range. Second, the actual value of the result is listed below the range in hyperlinked text. Third, clicking on the hyperlinked value generates a pop-up screen that provides a brief

description of how the test value relates to the patient's physiology. If the result is abnormal, the pop-up screen also provides information on recommendations for lifestyle or medication changes that may reduce one's risk.

Visual[13,20,24]

Component description[13,18,22,23]

Appendix 2

The vignette used to provide patients with context for why they are using the prototype and to orient them to the task they needed to accomplish during the testing session:

> Your friend recently went to a doctor's appointment to see if he should start taking medication to lower his cholesterol. He received a notification from the doctor that he has three test results ready to view: a lipid panel, a hepatitis B test, and a liver function test. Unfortunately, he is out of town and doesn't have access to a computer to view his results. Since your friend wants to know what his test results say, he gives you full permission to log into his account to view his lab results.

Appendix 3

Post-Session Questionnaire

1. What would you do after viewing these test results? **[text response]**
    a. **If they don't provide explanation of reason for their plan of action, ask:
        i. Why?
2. Was there any information missing?
3. Was there too much information?
4. Did you find yourself wanting to "Google" anything about you test results?
5. Rate your confidence level in your understanding of each test result, 1 being not at all confident, and 5 being very confident:

Lipid profile (cholesterol):

[Not at all confident 1 2 3 4 5 Very confident]

Liver function test:

[Not at all confident 1 2 3 4 5 Very confident]

Hepatitis B test:

[Not at all confident 1 2 3 4 5 Very confident]

i. If confidence is low or lower on one test compared to the others, ask them:

b. Why is your confidence less for that test? **[text response]**

6. Was there anything confusing or unclear about your test results? **[text response]**

7. Did you want to know more about your test result? **[yes / no]**

a. If so, what? **[text response]**

8. What was your **least favorite** thing about the prototype you just used? **[text response]**

9. What was your **favorite** thing about the prototype you just used? **[text response]**

10. Do you have any suggestions on how to improve the patient portal? **[text response]**

Reference

37. Healthwise. https://www.healthwise.org/ 2017; Accessed 5/1/2017.
